# 4-[5-(4-Chloro­phen­yl)-3-methyl-1*H*-pyrazol-1-yl]benzene­sulfonamide

**DOI:** 10.1107/S1600536813002134

**Published:** 2013-01-26

**Authors:** Muhammad A. Farrukh, Shaaban K. Mohamed, Maqsood Ahmed, Adel A. Marzouk, Samir M. El-Moghazy

**Affiliations:** a Department of Chemistry, Government College University, Lahore 54000, Pakistan; b Chemistry and Environmental Division, Manchester Metropolitan University, Manchester, M1 5GD, England; c Chemistry Department, Faculty of Science, Minia University, El-Minia, Egypt; d Pharmaceutical Chemistry Department, Faculty of Pharmacy, Al Azhar University, Egypt; e Faculty of Pharmacy, Pharmaceutical Chemistry Department, Cairo University, Cairo, Egypt

## Abstract

In the title compound, C_16_H_14_ClN_3_O_2_S, the dihedral angle between the benzene and pyrazole rings is 52.75 (2)°, while that between the pyrazole and 4-chloro­phenyl rings is 54.0 (3)°. The terminal sulfonamide group adopts an approximately tetra­hedral geometry about the S atom with a C—S—N angle of 108.33 (10)°. In the crystal, pairs of N—H⋯N hydrogen bonds lead to the formation of inversion dimers. These dimers are linked *via* a second pair of N—H⋯N hydrogen bonds and C—H⋯O interactions, forming a two-dimensional network lying parallel to the *bc* plane. The two-dimensional networks are linked *via* C—H⋯Cl interactions, forming a three-dimensional structure.

## Related literature

For the use of pyrazoles in metal-organic chemistry, see: Mukherjee (2000 ▶); Halcrow (2009 ▶). For the synthesis and pharmaceutical applications of pyrazole compounds, see, for example: Ranatunge *et al.* (2004 ▶); Szabo *et al.* (2008 ▶); Bekhit & Abdel-Aziem (2004 ▶); Bekhit *et al.* (2006 ▶); Rostom *et al.* (2003 ▶); Gökhan-Kelekçi *et al.* (2007 ▶); Lin *et al.* (2007 ▶); El-Moghazy *et al.* (2012 ▶); Sakya *et al.* (2008 ▶); Shen *et al.* (2004 ▶).
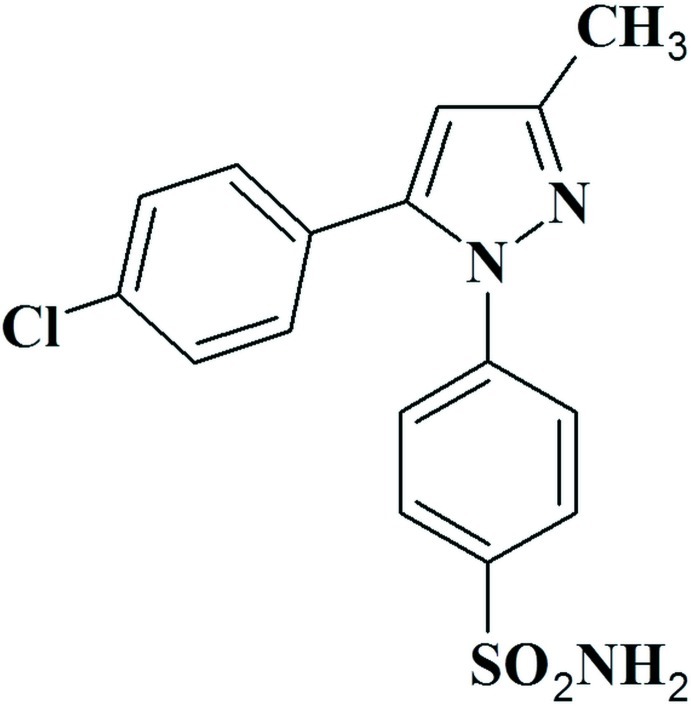


## Experimental

### 

#### Crystal data

C_16_H_14_ClN_3_O_2_S
*M* *_r_* = 347.81
Monoclinic, 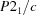

*a* = 15.878 (5) Å
*b* = 8.209 (5) Å
*c* = 12.953 (5) Å
β = 91.016 (5)°
*V* = 1688.1 (13) Å^3^
*Z* = 4
Mo *K*α radiation
μ = 0.36 mm^−1^
*T* = 296 K
0.33 × 0.32 × 0.18 mm

#### Data collection

Bruker Kappa APEXII CCD diffractometer
Absorption correction: analytical (*SADABS*; Bruker, 2009 ▶) *T*_min_ = 0.890, *T*_max_ = 0.938
17257 measured reflections
3462 independent reflections
2594 reflections with *I* > 2σ(*I*)
*R*_int_ = 0.030

#### Refinement

*R*[*F* ^2^ > 2σ(*F* ^2^)] = 0.041
*wR*(*F* ^2^) = 0.115
*S* = 1.03
3462 reflections
217 parameters
H atoms treated by a mixture of independent and constrained refinement
Δρ_max_ = 0.34 e Å^−3^
Δρ_min_ = −0.33 e Å^−3^

### 

Data collection: *APEX2* (Bruker, 2009 ▶); cell refinement: *SAINT* (Bruker, 2009 ▶); data reduction: *SAINT*; program(s) used to solve structure: *SIR92* (Altomare *et al.*, 1993 ▶); program(s) used to refine structure: *SHELXL97* (Sheldrick, 2008 ▶); molecular graphics: *ORTEP-3* (Farrugia, 2012 ▶) and *Mercury* (Macrae *et al.*, 2008 ▶); software used to prepare material for publication: *publCIF* (Westrip, 2010 ▶).

## Supplementary Material

Crystal structure: contains datablock(s) I, global_Publ_Block. DOI: 10.1107/S1600536813002134/sj5296sup1.cif

Structure factors: contains datablock(s) I. DOI: 10.1107/S1600536813002134/sj5296Isup2.hkl

Supplementary material file. DOI: 10.1107/S1600536813002134/sj5296Isup3.cml

Additional supplementary materials:  crystallographic information; 3D view; checkCIF report

## Figures and Tables

**Table 1:** Hydrogen-bond geometry (Å, °)

*D*—H⋯*A*	*D*—H	H⋯*A*	*D*⋯*A*	*D*—H⋯*A*
N1—H2*N*1⋯N3^i^	0.82 (2)	2.20 (2)	3.010 (3)	169 (2)
N1—H1*N*1⋯N3^ii^	0.84 (3)	2.33 (3)	3.157 (3)	167 (3)
C5—H5⋯O2^iii^	0.93	2.48	3.169	131
C3—H3⋯Cl1^iv^	0.93	2.93	3.602	130

Symmetry codes: (i) 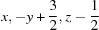; (ii) 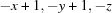; (iii) 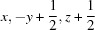; (iv) 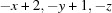.

**Fig. 1.**
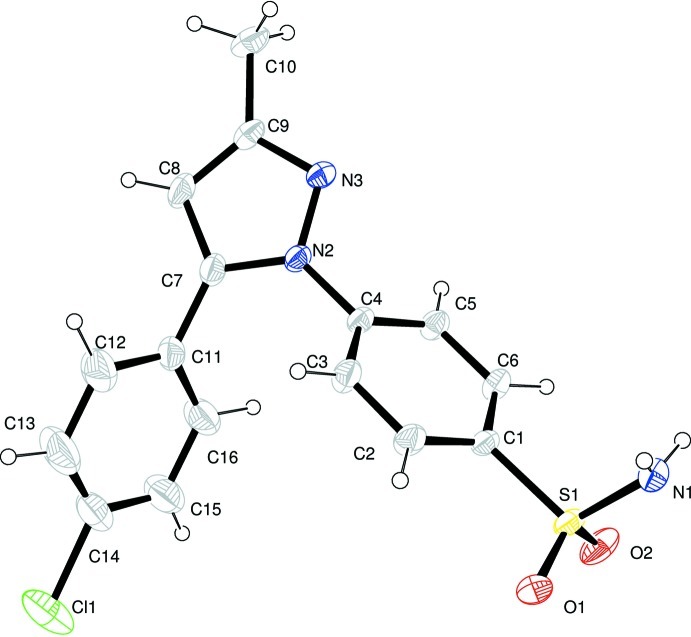
An ORTEPIII diagram of the molecule showing the atom numbering scheme and thermal ellipsoids drawn at the 50% probability level.

**Fig. 2.**
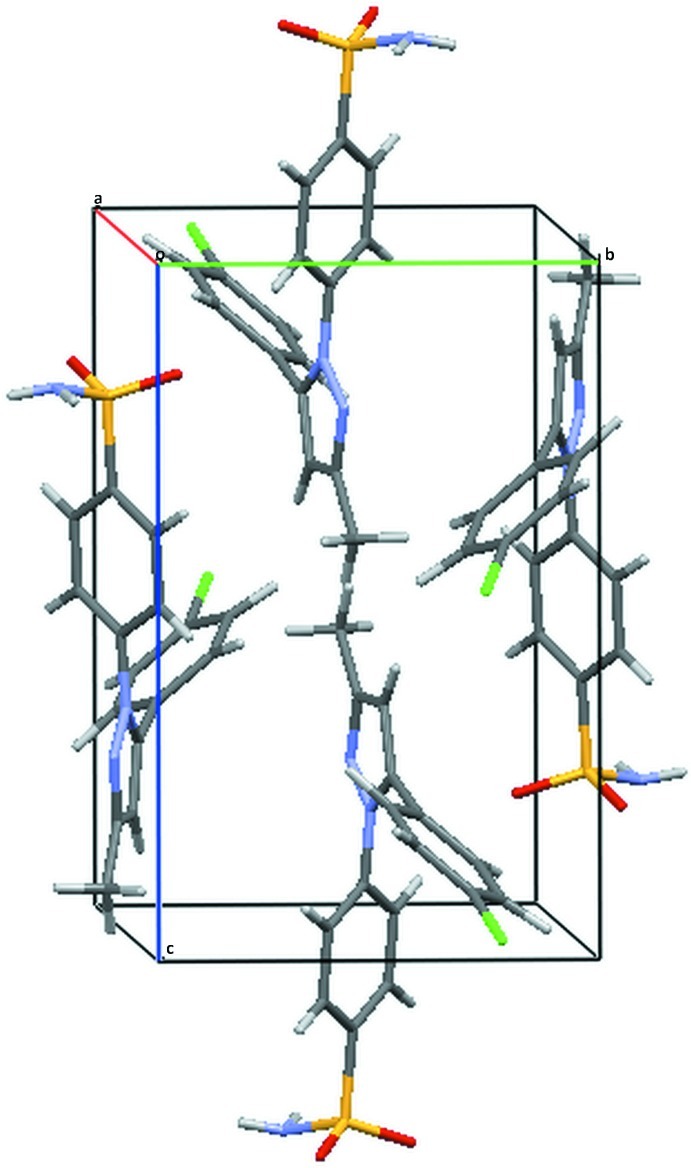
A view of the molecular packing along the a axis.

**Fig. 3.**
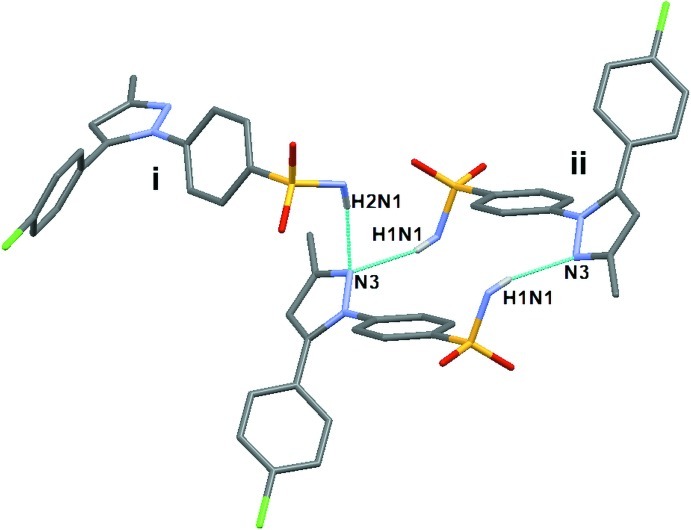
A trimer of molecules formed by intermolecular N–H···N hydrogen bonds. Symmetry codes: (i) x, -y + 3/2, z - 1/2; (ii) -x + 1, -y + 1, -z

**Fig. 4.**
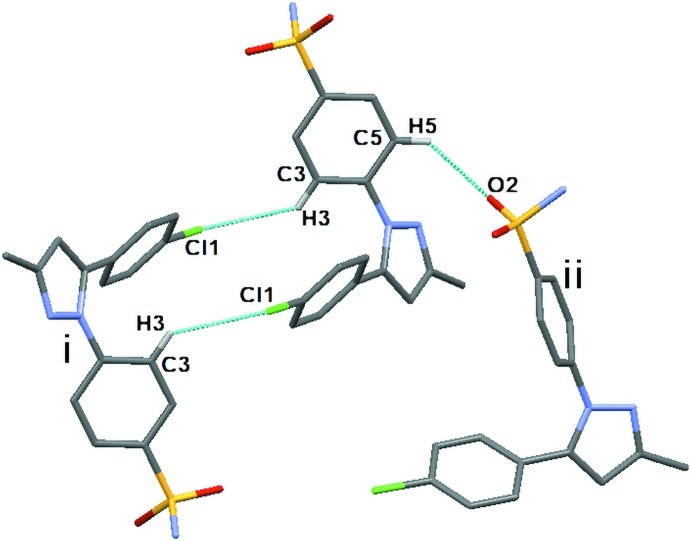
Image showing the intermolecular C–H···O and C–H···Cl hydrogen bonds. The H atoms not involved in any interaction have been omitted for clarity. Symmetry codes: (i) 2 - x, 1 - y, -z; (ii) x, 1/2 - y,1/2 + z]

## supplementary crystallographic information

### Comment

Pyrazoles and related compounds are common molecules used in coordination or organometallic chemistry as bridging ligands, utilizing the ring positions of the two N atoms (Mukherjee, 2000; Halcrow, 2009). In addition, pyrazole derivatives represent an important class of biologically and pharmacologically active molecules. Several pyrazole compounds have been reported to be potential therapeutic agents for the treatment of inflammation (Ranatunge *et al.*, 2004; Szabo *et al.*, 2008; Bekhit & Abdel-Aziem 2004; Bekhit *et al.*, 2006) including the marketed selective COX-2 drug, Celecoxib, that have been shown to be well tolerated with reduced gastrointestinal side effects (Sakya *et al.*, 2008). Moreover, various substituted pyrazoles were reported to possess antitumor properties (Rostom *et al.*, 2003; Lin *et al.*, 2007). Other pyrazoles were used for treating Alzheimer's disease (Gökhan-Kelekçi *et al.*, 2007) and acquired immunodeficiency syndrome (AIDS) (Shen *et al.*, 2004; El-Moghazy *et al.*, 2012).

The molecular assembly is built on the basis of intermolecular N—H···N type hydrogen bonds. The N3 atom on one side accepts a H1N1 atom from a neighbouring atom at a distance of 2.33 (3) Å and also accepts a H2N1 atom from a molecule on the opposite side at a distance of 2.20 (2) Å. There is a weak C–H···O type intermolecular hydrogen bond where the C5 atom donates its H atom to the O2 of the sulfonamide group at a distance of 2.48 Å and C—H—O angle of 130.8°. The molecule is also involved in the formation of a pair of weak intermolecular inversion related C3—H3···Cl1 hydrogen bonds where in one case the Cl1 atom accepts a H atom from a neighbouring molecule while in return, the C3 atom donates its H3 atom to the same molecule. The C–H···Cl distance in both cases is 2.93 Å and C—H—Cl angle is 130.0°.

### Experimental

A mixture of 1 mmol (197 mg) 1-(4-chlorophenyl)butane-1,3-dione, 1 mmol (224 mg) 4-hydrazinylbenzenesulfonamide hydrochloride, 82 mg sodium acetate and 60 mg glacial acetic acid in 50 ml e thanol was stirred at room temperature for 24 h. The mixture was filtered off and the filtrate was concentrated under vacuum to deposit the solid product which was collected, dried and recrystallized from ethanol to afford a very good yield (80%) of high quality crystals suitable for X-ray diffraction (498 – 499 K).

### Refinement

The H atoms attached to N1 were located in a difference map and were refined freely. All the H atoms attached to aromatic carbon atoms were initially located in the difference map but subsequently refined with a distance restraint of 0.93 Å and *U*iso(H) = 1.2*U*eq(C). The H atoms attached to C10 atom were positioned geometrically at idealized positions for methyl and were refined with C—H distance of 0.96 Å and *U*iso(H) = 1.5*U*eq(C).

### Crystal data

**Table:**

C_16_H_14_ClN_3_O_2_S	*F*(000) = 720
*M**_r_* = 347.81	*D*_x_ = 1.369 Mg m^−^^3^
Monoclinic, *P*2_1_/*c*	Mo *K*α radiation, λ = 0.71073 Å
Hall symbol: -P 2ybc	Cell parameters from 133 reflections
*a* = 15.878 (5) Å	θ = 2.7–26.0°
*b* = 8.209 (5) Å	µ = 0.36 mm^−^^1^
*c* = 12.953 (5) Å	*T* = 296 K
β = 91.016 (5)°	Block, colourless
*V* = 1688.1 (13) Å^3^	0.33 × 0.32 × 0.18 mm
*Z* = 4	

### Data collection

**Table:**

Bruker Kappa APEXII CCD diffractometer	3462 independent reflections
Radiation source: fine-focus sealed tube	2594 reflections with *I* > 2σ(*I*)
Graphite monochromator	*R*_int_ = 0.030
ω and φ scans	θ_max_ = 26.4°, θ_min_ = 2.8°
Absorption correction: analytical (*SADABS*; Bruker, 2009)	*h* = −19→19
*T*_min_ = 0.890, *T*_max_ = 0.938	*k* = −10→10
17257 measured reflections	*l* = −16→16

### Refinement

**Table:**

Refinement on *F*^2^	Primary atom site location: structure-invariant direct methods
Least-squares matrix: full	Secondary atom site location: difference Fourier map
*R*[*F*^2^ > 2σ(*F*^2^)] = 0.041	Hydrogen site location: inferred from neighbouring sites
*wR*(*F*^2^) = 0.115	H atoms treated by a mixture of independent and constrained refinement
*S* = 1.03	*w* = 1/[σ^2^(*F*_o_^2^) + (0.0506*P*)^2^ + 0.8701*P*] where *P* = (*F*_o_^2^ + 2*F*_c_^2^)/3
3462 reflections	(Δ/σ)_max_ < 0.001
217 parameters	Δρ_max_ = 0.34 e Å^−^^3^
0 restraints	Δρ_min_ = −0.33 e Å^−^^3^

### Special details

**Table:**

Geometry. All e.s.d.'s (except the e.s.d. in the dihedral angle between two l.s. planes) are estimated using the full covariance matrix. The cell e.s.d.'s are taken into account individually in the estimation of e.s.d.'s in distances, angles and torsion angles; correlations between e.s.d.'s in cell parameters are only used when they are defined by crystal symmetry. An approximate (isotropic) treatment of cell e.s.d.'s is used for estimating e.s.d.'s involving l.s. planes.
Refinement. Refinement of *F*^2^ against ALL reflections. The weighted *R*-factor *wR* and goodness of fit *S* are based on *F*^2^, conventional *R*-factors *R* are based on *F*, with *F* set to zero for negative *F*^2^. The threshold expression of *F*^2^ > σ(*F*^2^) is used only for calculating *R*-factors(gt) *etc*. and is not relevant to the choice of reflections for refinement. *R*-factors based on *F*^2^ are statistically about twice as large as those based on *F*, and *R*- factors based on ALL data will be even larger.

### Fractional atomic coordinates and isotropic or equivalent isotropic displacement parameters (Å^2^)

**Table:**

	*x*	*y*	*z*	*U*_iso_*/*U*_eq_	
S1	0.62888 (4)	0.52544 (6)	−0.26035 (4)	0.03590 (16)	
Cl1	1.07396 (5)	0.23198 (14)	0.03417 (10)	0.1065 (4)	
O1	0.70283 (10)	0.5918 (2)	−0.30442 (11)	0.0545 (5)	
N1	0.55446 (14)	0.6528 (2)	−0.27978 (14)	0.0381 (4)	
N3	0.62419 (11)	0.5109 (2)	0.25734 (12)	0.0338 (4)	
O2	0.59721 (12)	0.37105 (18)	−0.29389 (11)	0.0551 (5)	
N2	0.68811 (10)	0.4740 (2)	0.19252 (12)	0.0327 (4)	
C1	0.64780 (13)	0.5101 (2)	−0.12544 (14)	0.0316 (4)	
C3	0.72856 (14)	0.5768 (3)	0.02534 (16)	0.0434 (5)	
H3	0.7738	0.6306	0.0567	0.052*	
C6	0.59263 (14)	0.4216 (3)	−0.06641 (15)	0.0383 (5)	
H6	0.5465	0.3703	−0.0975	0.046*	
C4	0.67440 (13)	0.4873 (2)	0.08368 (14)	0.0311 (4)	
C11	0.83783 (14)	0.3838 (3)	0.19450 (17)	0.0467 (6)	
C2	0.71592 (14)	0.5872 (3)	−0.08065 (16)	0.0429 (5)	
H2	0.7532	0.6457	−0.1210	0.051*	
C5	0.60608 (13)	0.4095 (3)	0.03862 (15)	0.0372 (5)	
H5	0.5694	0.3493	0.0788	0.045*	
C9	0.65766 (15)	0.4978 (3)	0.35195 (16)	0.0403 (5)	
C10	0.60555 (18)	0.5323 (3)	0.44406 (17)	0.0550 (7)	
H10A	0.5507	0.5687	0.4219	0.082*	
H10B	0.6322	0.6156	0.4851	0.082*	
H10C	0.6002	0.4349	0.4844	0.082*	
C7	0.76017 (14)	0.4366 (3)	0.24501 (16)	0.0415 (5)	
C16	0.83766 (17)	0.2553 (4)	0.1266 (3)	0.0688 (8)	
H16	0.7877	0.1994	0.1130	0.083*	
C8	0.74152 (15)	0.4516 (3)	0.34751 (17)	0.0506 (6)	
H8	0.7781	0.4341	0.4033	0.061*	
C14	0.98314 (17)	0.2900 (4)	0.0978 (3)	0.0676 (8)	
C12	0.91258 (18)	0.4628 (4)	0.2135 (3)	0.0808 (10)	
H12	0.9143	0.5492	0.2599	0.097*	
C15	0.91033 (19)	0.2080 (4)	0.0784 (3)	0.0800 (10)	
H15	0.9095	0.1204	0.0329	0.096*	
C13	0.98526 (19)	0.4160 (5)	0.1648 (3)	0.0943 (12)	
H13	1.0355	0.4710	0.1781	0.113*	
H2N1	0.5670 (16)	0.747 (3)	−0.266 (2)	0.050 (8)*	
H1N1	0.5061 (18)	0.620 (4)	−0.265 (2)	0.061 (9)*	

### Atomic displacement parameters (Å^2^)

**Table:**

	*U*^11^	*U*^22^	*U*^33^	*U*^12^	*U*^13^	*U*^23^
S1	0.0498 (3)	0.0367 (3)	0.0213 (2)	0.0057 (2)	0.0010 (2)	−0.0004 (2)
Cl1	0.0537 (5)	0.1252 (8)	0.1417 (9)	0.0173 (5)	0.0359 (5)	0.0154 (7)
O1	0.0500 (10)	0.0833 (12)	0.0304 (8)	0.0055 (9)	0.0090 (7)	0.0095 (8)
N1	0.0445 (12)	0.0344 (10)	0.0352 (10)	−0.0008 (9)	−0.0052 (8)	0.0011 (8)
N3	0.0408 (10)	0.0353 (9)	0.0254 (8)	0.0006 (7)	0.0019 (7)	0.0009 (7)
O2	0.0967 (14)	0.0358 (8)	0.0324 (8)	0.0054 (9)	−0.0079 (8)	−0.0078 (7)
N2	0.0359 (9)	0.0379 (9)	0.0241 (8)	−0.0004 (7)	−0.0012 (7)	0.0010 (7)
C1	0.0395 (11)	0.0323 (10)	0.0229 (9)	0.0057 (9)	0.0007 (8)	0.0003 (8)
C3	0.0409 (12)	0.0568 (14)	0.0322 (11)	−0.0150 (10)	−0.0038 (9)	0.0033 (10)
C6	0.0434 (12)	0.0406 (11)	0.0305 (10)	−0.0086 (10)	−0.0058 (9)	0.0012 (9)
C4	0.0369 (11)	0.0330 (10)	0.0235 (9)	0.0028 (8)	−0.0006 (8)	0.0011 (8)
C11	0.0370 (12)	0.0625 (15)	0.0405 (12)	0.0009 (11)	−0.0062 (10)	0.0068 (11)
C2	0.0423 (12)	0.0556 (14)	0.0309 (11)	−0.0108 (11)	0.0035 (9)	0.0075 (10)
C5	0.0419 (12)	0.0399 (11)	0.0297 (10)	−0.0081 (9)	0.0004 (9)	0.0046 (9)
C9	0.0529 (14)	0.0426 (12)	0.0255 (10)	−0.0081 (10)	−0.0005 (9)	0.0010 (9)
C10	0.0704 (17)	0.0659 (16)	0.0290 (11)	−0.0104 (13)	0.0082 (11)	−0.0044 (11)
C7	0.0388 (12)	0.0524 (13)	0.0331 (11)	−0.0018 (10)	−0.0069 (9)	0.0033 (10)
C16	0.0398 (14)	0.078 (2)	0.089 (2)	−0.0038 (13)	0.0061 (14)	−0.0206 (17)
C8	0.0510 (14)	0.0721 (17)	0.0284 (11)	−0.0033 (12)	−0.0110 (10)	0.0048 (11)
C14	0.0388 (14)	0.083 (2)	0.081 (2)	0.0101 (14)	0.0075 (14)	0.0159 (17)
C12	0.0460 (16)	0.102 (2)	0.095 (2)	−0.0116 (16)	−0.0052 (16)	−0.027 (2)
C15	0.0517 (18)	0.088 (2)	0.100 (3)	0.0071 (16)	0.0087 (17)	−0.0284 (19)
C13	0.0375 (16)	0.111 (3)	0.134 (3)	−0.0149 (17)	0.0036 (18)	−0.018 (3)

### Geometric parameters (Å, º)

**Table:**

S1—O1	1.4229 (17)	C11—C16	1.373 (4)
S1—O2	1.4284 (18)	C11—C7	1.471 (3)
S1—N1	1.594 (2)	C2—H2	0.9300
S1—C1	1.772 (2)	C5—H5	0.9300
Cl1—C14	1.740 (3)	C9—C8	1.387 (3)
N1—H2N1	0.82 (3)	C9—C10	1.491 (3)
N1—H1N1	0.84 (3)	C10—H10A	0.9600
N3—C9	1.331 (3)	C10—H10B	0.9600
N3—N2	1.363 (2)	C10—H10C	0.9600
N2—C7	1.356 (3)	C7—C8	1.371 (3)
N2—C4	1.427 (2)	C16—C15	1.378 (4)
C1—C2	1.373 (3)	C16—H16	0.9300
C1—C6	1.380 (3)	C8—H8	0.9300
C3—C4	1.369 (3)	C14—C13	1.351 (5)
C3—C2	1.387 (3)	C14—C15	1.357 (4)
C3—H3	0.9300	C12—C13	1.379 (4)
C6—C5	1.377 (3)	C12—H12	0.9300
C6—H6	0.9300	C15—H15	0.9300
C4—C5	1.380 (3)	C13—H13	0.9300
C11—C12	1.371 (4)		
			
O1—S1—O2	120.40 (11)	C6—C5—H5	120.3
O1—S1—N1	107.47 (12)	C4—C5—H5	120.3
O2—S1—N1	106.13 (12)	N3—C9—C8	110.59 (19)
O1—S1—C1	107.23 (10)	N3—C9—C10	120.2 (2)
O2—S1—C1	106.82 (9)	C8—C9—C10	129.2 (2)
N1—S1—C1	108.33 (10)	C9—C10—H10A	109.5
S1—N1—H2N1	114.2 (18)	C9—C10—H10B	109.5
S1—N1—H1N1	116 (2)	H10A—C10—H10B	109.5
H2N1—N1—H1N1	118 (3)	C9—C10—H10C	109.5
C9—N3—N2	105.06 (17)	H10A—C10—H10C	109.5
C7—N2—N3	111.87 (16)	H10B—C10—H10C	109.5
C7—N2—C4	128.71 (18)	N2—C7—C8	105.7 (2)
N3—N2—C4	119.31 (16)	N2—C7—C11	123.38 (19)
C2—C1—C6	120.78 (18)	C8—C7—C11	130.8 (2)
C2—C1—S1	120.16 (16)	C11—C16—C15	121.0 (3)
C6—C1—S1	119.06 (15)	C11—C16—H16	119.5
C4—C3—C2	119.88 (19)	C15—C16—H16	119.5
C4—C3—H3	120.1	C7—C8—C9	106.77 (19)
C2—C3—H3	120.1	C7—C8—H8	126.6
C5—C6—C1	119.78 (19)	C9—C8—H8	126.6
C5—C6—H6	120.1	C13—C14—C15	120.7 (3)
C1—C6—H6	120.1	C13—C14—Cl1	120.2 (2)
C3—C4—C5	120.80 (18)	C15—C14—Cl1	119.2 (3)
C3—C4—N2	120.02 (18)	C11—C12—C13	121.0 (3)
C5—C4—N2	119.18 (18)	C11—C12—H12	119.5
C12—C11—C16	118.0 (3)	C13—C12—H12	119.5
C12—C11—C7	120.7 (2)	C14—C15—C16	119.6 (3)
C16—C11—C7	121.3 (2)	C14—C15—H15	120.2
C1—C2—C3	119.3 (2)	C16—C15—H15	120.2
C1—C2—H2	120.4	C14—C13—C12	119.7 (3)
C3—C2—H2	120.4	C14—C13—H13	120.2
C6—C5—C4	119.44 (19)	C12—C13—H13	120.2
			
C9—N3—N2—C7	0.7 (2)	N2—N3—C9—C10	179.11 (19)
C9—N3—N2—C4	−175.84 (17)	N3—N2—C7—C8	−0.5 (2)
O1—S1—C1—C2	12.9 (2)	C4—N2—C7—C8	175.7 (2)
O2—S1—C1—C2	143.19 (18)	N3—N2—C7—C11	176.7 (2)
N1—S1—C1—C2	−102.9 (2)	C4—N2—C7—C11	−7.2 (3)
O1—S1—C1—C6	−167.96 (17)	C12—C11—C7—N2	126.0 (3)
O2—S1—C1—C6	−37.6 (2)	C16—C11—C7—N2	−53.5 (4)
N1—S1—C1—C6	76.33 (19)	C12—C11—C7—C8	−57.6 (4)
C2—C1—C6—C5	−0.3 (3)	C16—C11—C7—C8	122.9 (3)
S1—C1—C6—C5	−179.52 (16)	C12—C11—C16—C15	−0.5 (5)
C2—C3—C4—C5	−1.3 (3)	C7—C11—C16—C15	179.0 (3)
C2—C3—C4—N2	178.9 (2)	N2—C7—C8—C9	0.1 (3)
C7—N2—C4—C3	−48.5 (3)	C11—C7—C8—C9	−176.8 (2)
N3—N2—C4—C3	127.4 (2)	N3—C9—C8—C7	0.4 (3)
C7—N2—C4—C5	131.7 (2)	C10—C9—C8—C7	−179.3 (2)
N3—N2—C4—C5	−52.5 (3)	C16—C11—C12—C13	0.8 (5)
C6—C1—C2—C3	−0.7 (3)	C7—C11—C12—C13	−178.7 (3)
S1—C1—C2—C3	178.52 (18)	C13—C14—C15—C16	0.9 (6)
C4—C3—C2—C1	1.5 (4)	Cl1—C14—C15—C16	−178.5 (3)
C1—C6—C5—C4	0.5 (3)	C11—C16—C15—C14	−0.3 (5)
C3—C4—C5—C6	0.3 (3)	C15—C14—C13—C12	−0.5 (6)
N2—C4—C5—C6	−179.87 (19)	Cl1—C14—C13—C12	178.9 (3)
N2—N3—C9—C8	−0.6 (2)	C11—C12—C13—C14	−0.3 (6)

### Hydrogen-bond geometry (Å, º)

**Table:**

*D*—H···*A*	*D*—H	H···*A*	*D*···*A*	*D*—H···*A*
N1—H2*N*1···N3^i^	0.82 (2)	2.20 (2)	3.010 (3)	169 (2)
N1—H1*N*1···N3^ii^	0.84 (3)	2.33 (3)	3.157 (3)	167 (3)
C5—H5···O2^iii^	0.93	2.48	3.169	131
C3—H3···Cl1^iv^	0.93	2.93	3.602	130

Symmetry codes: (i) *x*, −*y*+3/2, *z*−1/2; (ii) −*x*+1, −*y*+1, −*z*; (iii) *x*, −*y*+1/2, *z*+1/2; (iv) −*x*+2, −*y*+1, −*z*.
